# The role of periostin (OSF-2) in the cytoadherence phenomena mediated by malaria parasites

**DOI:** 10.3389/fcimb.2025.1599872

**Published:** 2025-05-13

**Authors:** Zhi-Ying Phong, Joo-Yie Chin, Yee Ling Ng, Nurul Izza Zakaria, Siti Nur Athirah-Azman, Varakorn Kosaisavee, Laurent Rénia, Wenn-Chyau Lee

**Affiliations:** ^1^ Department of Parasitology, Faculty of Medicine, Universiti Malaya, Kuala Lumpur, Malaysia; ^2^ Department of Medical Microbiology, Faculty of Medicine, Universiti Malaya, Kuala Lumpur, Malaysia; ^3^ Collaborative Drug Discovery Research (CDDR) Group, Faculty of Pharmacy, Universiti Teknologi MARA (UiTM), Bandar Puncak Alam, Selangor, Malaysia; ^4^ Department of Pathology, Hospital Tengku Ampuan Afzan, Kuantan, Pahang, Malaysia; ^5^ Higher Institution Centre of Excellence, Tropical Infectious Diseases Research and Education Centre (TIDREC), Universiti Malaya, Kuala Lumpur, Malaysia; ^6^ Institute for Advanced Studies, Universiti Malaya, Kuala Lumpur, Malaysia; ^7^ Department of Parasitology and Entomology, Faculty of Public Health, Mahidol University, Bangkok, Thailand; ^8^ Lee Kong Chian School of Medicine, Nanyang Technological University (NTU), Singapore, Singapore; ^9^ School of Biological Sciences, Nanyang Technological University (NTU), Singapore, Singapore; ^10^ A*STAR Infectious Diseases Labs, Agency for Science, Technology and Research (A*STAR), Singapore, Singapore

**Keywords:** *Plasmodium falciparum*, *Plasmodium knowlesi*, OSF-2, endothelial binding, rosetting

## Abstract

**Introduction:**

The pathogenesis of severe malaria is primarily attributed to the cytoadherence properties of *Plasmodium*-infected erythrocytes (IRBC), which include rosetting and IRBC-endothelial cytoadherence. These cytoadherence events are influenced by various parasite- and host-derived factors. Previously, antibodies against human periostin (OSF-2), an inflammation-associated protein, were reported to inhibit rosetting. In this study, we aimed to characterize the OSF-2-mediated cytoadherence in infections caused by *Plasmodium falciparum* (the most fatal human malaria parasite) and *P. knowlesi* (an emerging, potentially fatal zoonotic malaria parasite).

**Methods:**

Laboratory-adapted *P. falciparum* and *P. knowlesi* isolates were cultured, and the late-stage parasites were purified for experiments using recombinant human OSF-2.

**Results:**

We found that OSF-2 at a concentration of 200 ng/ml induced rosette-stimulation in both parasite species. Furthermore, we demonstrated the serum dependency of OSF-2-mediated rosetting. The rosette-stimulating effect of OSF-2 was completely abolished when IRBC were treated with a low concentration of trypsin. This suggests a role for *P. falciparum* erythrocyte membrane protein 1 (PfEMP1) in OSF-2-mediated rosetting by *P. falciparum*, and reveals the trypsin-sensitive nature of the P. knowlesi-derived ligands involved in OSF-2-mediated rosetting. We also found that OSF-2-mediated rosetting was independent of the ABO blood group. Additionally, we demonstrated the ability of OSF-2 to disrupt the IRBC-endothelial binding.

**Discussion:**

This work contributes to our understanding of the host-parasite interactions in malaria pathobiology.

## 1 Introduction

Malaria remains a significant global health concern, particularly in tropical and subtropical regions (WHO, 2024). Among the medically important malaria parasites, *Plasmodium falciparum* is the primary cause of the malaria-associated fatalities worldwide (WHO, 2024). Since the beginning of the third millennium, the frequent reporting of potentially fatal zoonotic malaria caused by *P. knowlesi* in Southeast Asia has led to its recognition as the fifth medically important malaria parasite (White, 2008; Chin et al., 2020; Lee et al., 2022a).

The pathogenesis of severe malaria is strongly associated with the cytoadherence properties of infected erythrocytes (IRBC) (Lee et al., 2019; 2022b). *P. falciparum*-IRBC with display of ‘knobs’ on their surface can adhere to endothelial cells via interactions with various endothelial surface receptors such as CD36, ICAM-1, VCAM-1, EPCR, P-selectin and E-selectin (Turner et al., 1994; Yipp et al., 2000; Armah et al., 2005; Metwally et al., 2017; Lee et al., 2019). This leads to the sequestration of late-stage IRBC in the deep vasculature (Roberts et al., 1985). This process triggers endothelial activation and inflammation, resulting in vascular damage and severe malaria, which can manifest as cerebral malaria, acute respiratory distress syndrome, acute kidney injury, placental malaria and severe malaria-induced anemia (Chilongola et al., 2009; Lee et al., 2019). Notably, most IRBC-endothelial cytoadherence studies have focused on *P*. *falciparum*, with fewer studies examining *P. vivax* and *P. knowlesi* (Sherman et al., 2003; Carvalho et al., 2010; Lee et al., 2022c). Furthermore, the clinical manifestations of severe complications vary among different species of malaria parasites. For example, neurological complications are predominantly associated with falciparum malaria (Trivedi and Chakravarty, 2022), and are rarely reported in knowlesi malaria patients (Cox-Singh et al., 2010; Kantele and Jokiranta, 2011). Therefore, findings from *P. falciparum* studies may not be directly applicable to *P. knowlesi* or other malaria parasites.

In addition to IRBC-endothelial binding, rosetting – the stable adherence of IRBC to uninfected erythrocytes (URBC), has been discovered as another related but independent IRBC cytoadherence phenomenon (David et al., 1988; Kaul et al., 1991). Rosetting has been reported in all medically important malaria parasites (Lowe et al., 1998; Lee et al., 2014, 2022). Similar to the IRBC-endothelial cytoadherence investigations, most rosetting studies have focused on *P. falciparum*. Notably, IRBC-endothelial binding and rosetting in *P. falciparum* shared many biological properties, including the involvement of similar parasite-derived ligands and host-derived receptors (Handunnetti et al., 1992; Chen et al., 1998; Chilongola et al., 2009; Bachmann et al., 2022; Rajan Raghavan et al., 2023), while the ligands and receptors involved in the cytoadherence mediated by other species of malaria parasites remain poorly understood. Importantly, the dynamics of IRBC cytoadherence to both endothelial cells and URBC have yet to be fully elucidated. Beyond parasite-derived ligands and host-derived receptors, these cytoadherence phenomena can be influenced by other host-derived factors such as complement factor D (CFD) and insulin-like growth factor binding protein 7 (IGFBP7) (Luginbühl et al., 2007; Lee et al., 2020). Previously, we observed that antibodies against human periostin (OSF-2) could block the rosette-stimulating effect induced by the culture supernatant of human monocytic THP-1 cells primed with *P. falciparum* antigens (Lee et al., 2020), suggesting a role for OSF-2 in IRBC-mediated cytoadherence during malaria pathogenesis.

Periostin, originally identified as osteoblast-specific factor-2 (OSF-2), was discovered as a potential cell adhesion protein for pre-osteoblasts in the mouse osteoblastic MC3T3-E1 cell line (Takeshita et al., 1993). Subsequently, it was named periostin due to its presence in the periosteum and periodontal ligament (Horiuchi et al., 1999). This protein interacts with various extracellular matrix proteins, and involves in regulating intercellular adhesion (Takayama et al., 2006). Notably, OSF-2 expression is associated with localized and systemic inflammatory conditions, including infections (Takayama et al., 2006; Sonnenberg-Riethmacher et al., 2021; Tuna et al., 2024), and its secretion by several immune cells is increased upon activation (Liu et al., 2014; Li et al., 2015). Given the association between OSF-2 and inflammation, as well as its potential involvement in IRBC-mediated cytoadherence events critical to malaria pathogenesis, the impact of OSF-2 on these phenomena warrants further investigation. Here, we characterized the effect of OSF-2 on rosetting and IRBC-endothelial cytoadherence in *P. falciparum* and *P. knowlesi*.

## 2 Materials and methods

### 2.1 Materials used

Information of materials used is available in **Supplementary Table 1**.

### 2.2 Study approval and general conditions of the experiments

The sample collection and all experiments were conducted under guidelines approved by the Institutional Biosafety and Biosafety Committee (IBBC) of Universiti Malaya (UMIBBC/PA/R/FOM/PARA-025/2022) and University of Malaya Medical Centre (UMMC) Medical Research Ethics Committee (MRECID#2024312-13526). All experiments in this study were conducted with at least six biological replicates unless stated otherwise, where biological replicates of parasite were defined as individual parasite cultures of different culture batches that contained distinct individual IRBC (either with or without distinct individual URBC). The biological replicates of endothelial cell lines were defined as independent batches of cultured cell lines seeded in distinct culture chambers of fixed dimensions and culture conditions.

### 2.3 Parasite and endothelial cell cultures

Laboratory-adapted *P. falciparum* and *P. knowlesi* were thawed using the sodium chloride method and cultured with group O RBC at 2% hematocrit in RPMI-1640 media enriched with AlbuMAX II and 20% (v/v) heat-inactivated human serum. Cultures were maintained under standard *in vitro* cultivation conditions: 37°C, humidity exceeding 90%, and gas mixture of 7% CO_2_, 5% O_2_, 90% N_2_ (as previously described in Lee et al., 2014 and Lee et al., 2022c). Late-stage parasites were purified using a magnetic-activated cell sorting (MACS) system.

For the cultivation of human endothelial cell lines, culture flask was treated with 0.5% gelatin solution for 6 hours at 37°C prior to the inoculation of thawed cell lines. The cell lines used were human cerebral microvascular endothelial cell line (hCMEC/d3), human renal glomerular endothelial cell line (HRGEC), human pulmonary microvascular endothelial cell line (HPMEC) and human umbilical vein endothelial cell line (HUVEC)]. They were cultured with the complete endothelial cell medium (ECM).

### 2.4 Recombinant periostin protein solution preparation

Recombinant human periostin protein (henceforth known as “OSF-2”) was reconstituted with 1X phosphate-buffered saline (PBS) (stock protein concentration: 100 μg/ml) according to the manufacturer’s manual. The stock was aliquoted and stored at 4°C until its subsequent use.

### 2.5 Rosetting characterization

The experiment was conducted when approximately 70% of the parasite population had reached late-stage development (late trophozoite to schizont stages). The parasite culture suspension was exposed to various working concentrations of OSF-2. A separate aliquot of parasite culture suspension, which was unexposed to OSF-2, served as the control. Notably, the selected OSF-2 concentrations were within the reported pathophysiologic range of serum OSF-2 levels (**Supplementary Table 2**) (Okamoto et al., 2011; Yamaguchi et al., 2013; Kou et al., 2014; Caswell-Smith et al., 2016; Yang et al., 2016; Zhu et al., 2016; Thuwajit et al., 2017; Ding et al., 2018; Gadermaier et al., 2018; Yildiz et al., 2021). The plate was incubated for one hour under standard *in vitro* cultivation conditions before the rosetting assay (Lee et al., 2013; Lee and Rénia, 2020). Briefly, the culture suspension was stained with Giemsa, transferred to a clean glass slide, and mounted with a glass cover-slip. The wet mount was then examined with a light microscope using immersion oil magnification. The rosetting rate was calculated as the percentage of IRBCs that formed rosettes, by recruiting 200 IRBC. After determining the optimal working concentration of OSF-2 for subsequent experiments, the importance of human serum availability to the OSF-2-mediated rosetting was tested by repeating the assay with the selected working concentration of OSF-2 in culture media supplied with different levels of human serum enrichment, where the original culture medium (20% serum-enriched medium) was removed via centrifugation (300 g for 2 minutes), and the pelleted cells were suspended with experimented culture media.

In a separate experiment, trypsinization was performed based on previous trypsin sensitivity profiling of *P. falciparum* cytoadherence ligands (Kyes et al., 2000; Niang et al., 2014). Briefly, purified late-stage IRBC were divided into three groups. One treated with 10 µg/mL of trypsin, another with 1mg/ml of trypsin, and a third untreated control group. Following a ten-minute incubation under *in vitro* culture conditions, the enzymatic reaction was stopped with human serum-enriched culture medium. Each group was then divided into two parts; one was supplemented with OSF-2 (200 ng/ml) and the other served as a control. The rosetting assay was conducted after one hour of incubation under *in vitro* cultivation conditions.

An additional experiment was conducted, where MACS-sorted late-stage IRBC were divided into four groups. Each group of purified IRBC was mixed with URBC of A, B, O, and AB groups, respectively, to create a suspension of 1% parasitemia with 2% hematocrit. Each group was further divided into two experiment fractions (OSF-2-exposed and unexposed control), and incubated under *in vitro* cultivation conditions for one hour before the rosetting assay.

### 2.6 IRBC-endothelial cytoadherence assay

When the cultures of endothelial cell lines reached approximately 70% confluency, they were transferred into the gelatin-coated eight-well LABTEK chamber slides (each well was seeded with 1 × 10^5^ cells). On the following day, a mixture of ECM and parasite antigen suspension in a 2:1 ratio was added to the seeded cell lines and incubated under *in vitro* cultivation conditions for 24 hours, to ‘prime’ the cells with parasite antigens (Lee et al., 2022c). After cell priming, each well of the seeded cells was allocated parasite culture suspension (1% parasitemia, 2% hematocrit) and OSF-2 suspension (200 ng/ml). An aliquot of suspension from each well was taken for rosetting assay. The endothelial cell-parasite mixture was incubated for two hours under *in vitro* cultivation conditions. Subsequently, the suspension from each well was collected into separate microcentrifuge tubes for rosetting assay to evaluate the before-after effect. For the seeded endothelial cells, the unbound IRBCs were gently washed away with human serum-enriched medium for three times. This was followed by fixation with ice-cold absolute methanol for ten minutes. The chambers were then removed from the chamber slides, and the slides were stained with 5% Giemsa for 20 minutes before being examined under a light microscope using immersion oil magnification. The IRBC–endothelial cell line cytoadherence rate was determined by counting the number of IRBCs attached to endothelial cells per 100 fields (equivalent to coverage of approximately 8,000 cells). The IRBC-endothelial cytoadherence assay was repeated with a separate set of “primed” cell lines that were not exposed to OSF-2 (as control).

### 2.7 Statistical analyses

GraphPad Prism 9.5.1 software was used for data analyses. Normality of the data was evaluated using Shapiro-Wilk test. For normally distributed datasets, Welch’s t-test was used for the comparison of two datasets with unequal variances. For paired comparisons, paired t-test was used. For multi-group comparison against a control group, One-way ANOVA with Dunnett’s test was performed. For non-normally distributed datasets, Mann-Whitney test was used to compare two datasets, and Wilcoxon matched-pairs signed rank test was used for paired comparison. For the comparison of multiple datasets, Kruskal-Wallis with Dunn’s test was conducted. For the comparison of multiple sets of matched non-parametric data, Friedman with Dunn’s test was performed. *P* values smaller than 0.05 were considered as statistically significant.

## 3 Results

### 3.1 Effect of OSF-2 on *Plasmodium* rosetting rates

A preliminary experiment was conducted to examine the basal rosetting rates of the parasites across seven cycles of cultivation post-thawing. Both species of parasites demonstrated persistent but fluctuating rosetting rates. The fluctuation of *P. knowlesi* rosetting rates across the monitored period was of insignificant difference (Kruskal-Wallis *H(2)* = 7.15; *P* = 0.41) (**Supplementary Figure 1A**). However, significant difference in rosetting rates of *P. falciparum* across the monitored cycles was found (Kruskal-Wallis *H(2)* = 16.11; *P* = 0.01) (**Supplementary Figure 1B**). Thus, culture cycle-matched statistical comparison was done for data collected from subsequent experiments.

**Figure 1:**
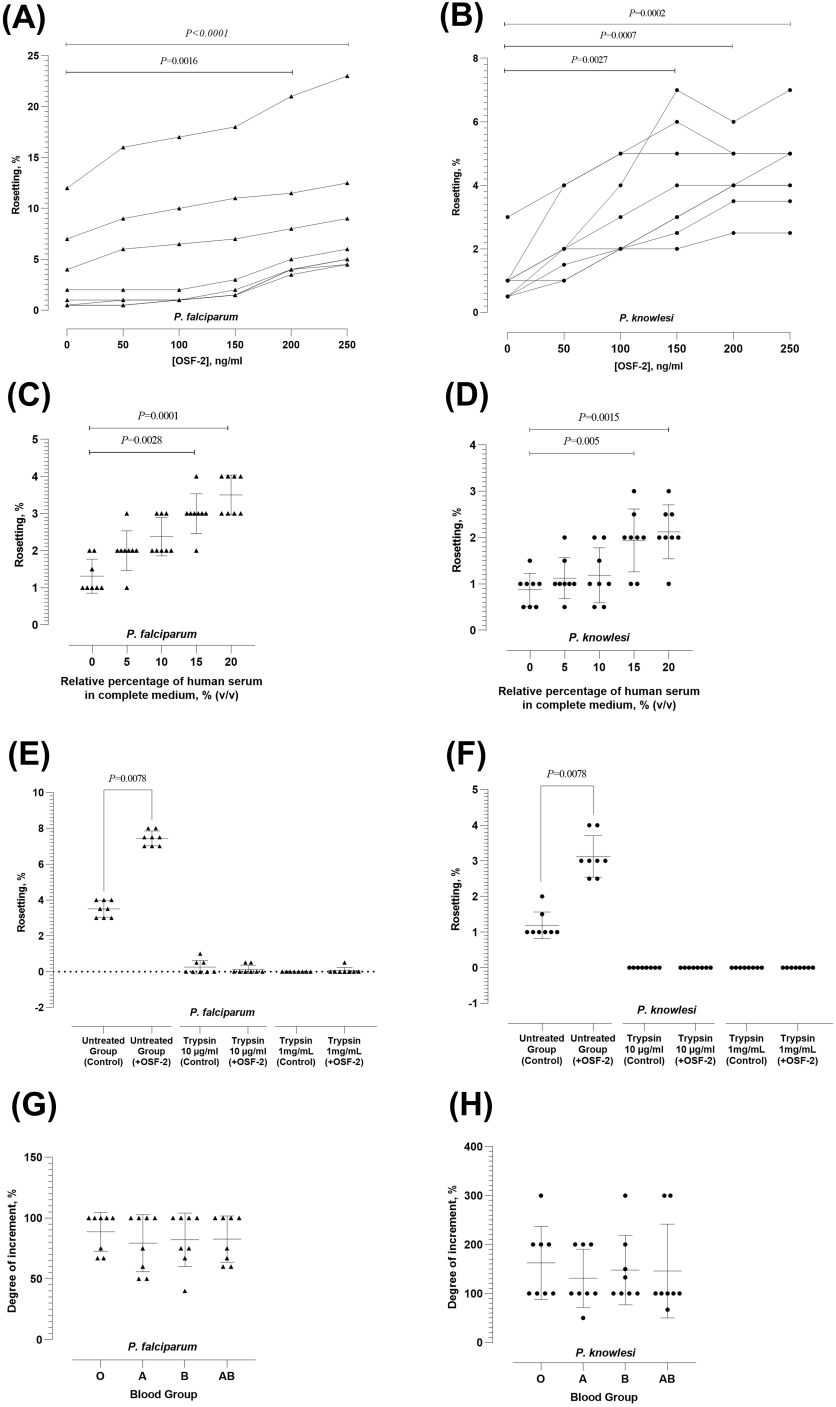
Characterization of OSF-2-mediated rosetting. **(A, B)** Rosetting rates of *P. falciparum* **(A)** and *P. knowlesi* **(B)** under different working concentrations of OSF-2. Dotted lines indicated data collected from the same parasite culture batch with experiments performed on the same day. **(C, D)** OSF-2-mediated rosetting of *P. falciparum* **(C)** and *P. knowlesi* **(D)** under conditions with different enrichment of human serum. **(E, F)** Effect of IRBC-trypsin treatment on OSF-2-mediated rosetting for *P. falciparum* **(E)** and *P. knowlesi* **(F)**. **(G, H)** Effect of human ABO blood groups on OSF-2-mediated rosetting for *P. falciparum* **(G)** and *P. knowlesi* **(H)**. Error bars in the plots represent mean and S.D.

The rosetting rates of *P. falciparum* and *P. knowlesi* responded positively to OSF-2 (Friedman with Dunn’s test *X^2^(7)* = 38.94; *P* < 0.0001 for *P. falciparum*; and *X^2^(7)* = 35.37; *P* < 0.0001 for *P. knowlesi*). For *P. falciparum*, significant rosette-stimulation was observed at OSF-2 working concentration of 200 ng/ml (*P* = 0.0016) and 250 ng/ml (*P* < 0.0001) (**Figure 1A**). For *P. knowlesi*, significant rosetting rate increment was recorded at OSF-2 working concentrations of 150 ng/ml (*P* = 0.0027), 200 ng/ml (*P* = 0.0007), and 250 ng/ml (*P* = 0.0002) (**Figure 1B**). Of note, there was no significant difference in rosette-stimulation between OSF-2 of 200 ng/ml and 250 ng/ml for sp*P. falciparum*. Similarly, no significant difference in *P. knowlesi* rosette-stimulation was found at OSF-2 of 150 ng/ml, 200 ng/ml and 250 ng/ml. Subsequent experiments were then conducted with 200 ng/ml OSF-2. The OSF-2-mediated rosetting was human serum-dependent for *P. falciparum* (Friedman with Dunn’s test *X^2^(7)* = 28.40; *P* < 0.0001) (**Figure 1C**) and *P. knowlesi* (Friedman with Dunn’s test *X^2^(7)* = 27.76; *P* < 0.0001 for *P. knowlesi*) (**Figure 1D**), where significant increment of rosetting rates was found at conditions supplied with 15% serum (*P* = 0.0028 and 0.005 for *P. falciparum* and *P. knowlesi*, respectively) and 20% serum (*P* = 0.0001 and 0.0015 for *P. falciparum* and *P. knowlesi*, respectively), as compared with the serum-free setting. For both species, the OSF-2-mediated rosette stimulation under conditions supplied with 15% and 20% serum were of insignificant difference. In short, OSF-2 stimulated *P. falciparum* and *P. knowlesi* to form more rosettes in a human serum-dependent manner.

### 3.2 Characterization of potential ligands and receptors involved in OSF*-*2-mediated rosetting

For *P. falciparum*, treatment with either low (10 µg/ml) or high (1 mg/ml) concentration of trypsin to IRBC abrogated the OSF-mediated rosette-stimulating effect (**Figure 1E**). Similarly, the OSF-2-mediated rosette-stimulation on *P. knowlesi* was abolished by the IRBC treatment with both concentrations of trypsin (**Figure 1F**). We also investigated the role of human ABO blood groups in OSF-2-mediated rosetting. The OSF-2-mediated rosetting in *P. falciparum* (**Figure 1G**) and *P. knowlesi* (**Figure 1H**) was independent of human ABO blood groups, where the OSF-2-mediated rosette-stimulation on IRBC purified from the same batch of parasite culture was insignificantly different when subjected to URBC of A, B, O and AB groups (Friedman with Dunn’s test *X^2^(7)* = 1.68; *P* = 0.64 for *P. falciparum*; and *X^2^(7)* = 3.16; *P* = 0.37 for *P. knowlesi*). Succinctly, the OSF-2 mediated rosetting by *P. falciparum* and *P. knowlesi* was trypsin-sensitive and independent of human blood ABO groups.

### 3.3 Effect of OSF-2 on the dynamic of rosetting and IRBC-endothelial cytoadherence

**Figure 2:**
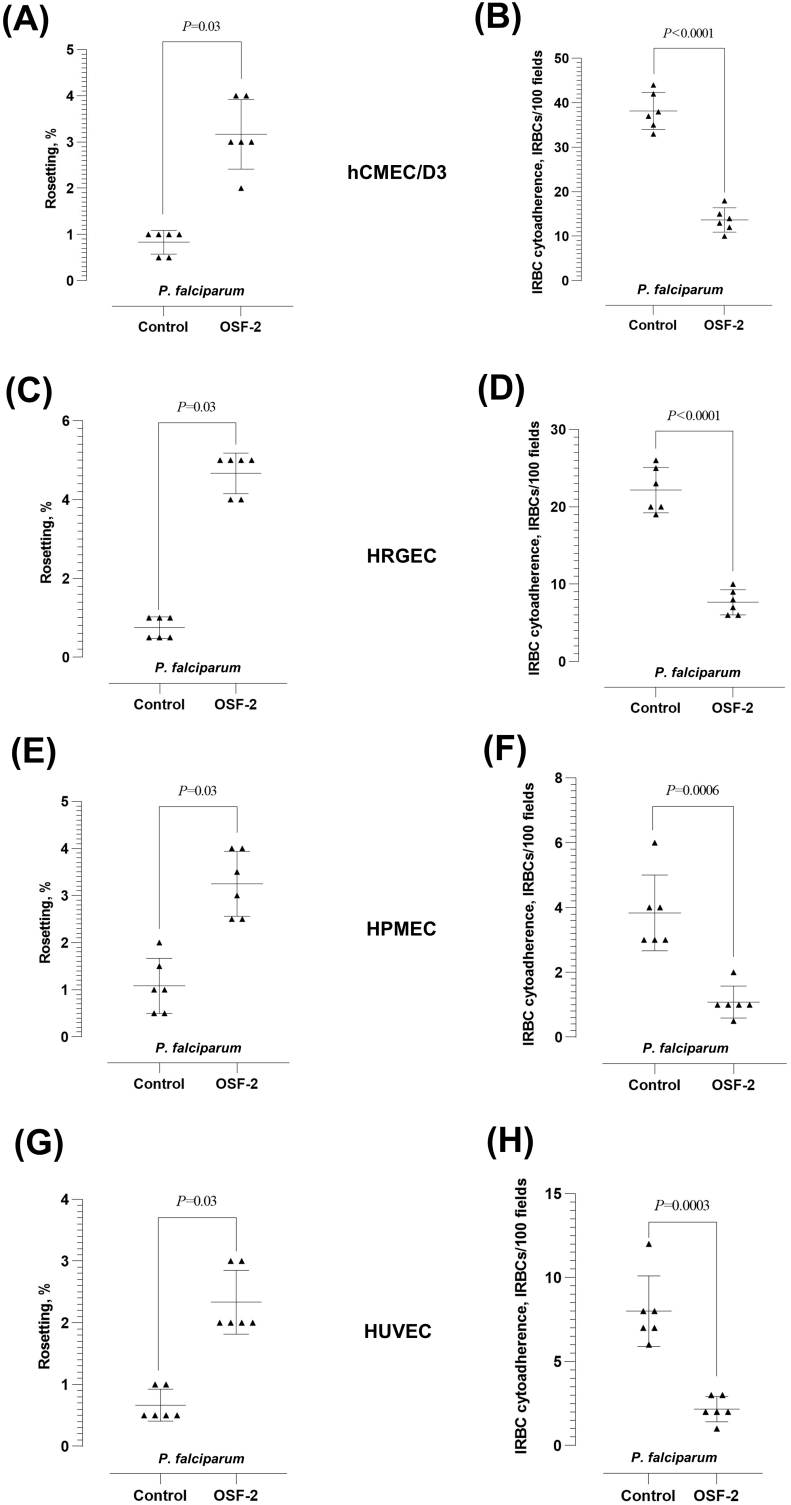
Effect of OSF-2 on the *P. falciparum-*IRBC cytoadherence dynamics between URBC and endothelial cells. **(A, B)** Rosetting rates **(A)** and IRBC-endothelial cytoadherence rates **(B)** from the experiments with hCMEC/D3. **(C, D)** Rosetting rates **(C)** and IRBC-endothelial cytoadherence rates **(D)** from the experiments with HRGEC. **(E, F)** Rosetting rates **(E)** and IRBC-endothelial cytoadherence rates **(F)** from the experiments with HPMEC. **(G, H)** Rosetting rates **(G)** and IRBC-endothelial cytoadherence rates **(H)** from the experiments with HUVEC. Error bars in the plots represent mean and S.D.

We evaluated the effect of OSF-2 on the *P. falciparum-*IRBC cytoadherence dynamics using endothelial cell lines hCMEC/d3 (**Figures 2A, B**), HRGEC (**Figures 2C, D**), HPMEC (**Figures 2E, F**), and HUVEC (**Figures 2G, H**). The protein demonstrated similar effects on the *P. falciparum-*IRBC cytoadherence dynamics with these endothelial cells. Following the parasite-endothelial cell line incubation, OSF-2 significantly increased the rosetting rates of *P. falciparum* as expected (Wilcoxon matched-pairs signed rank test *W* = 21; *P* = 0.03 for hCMEC/D3, HRGEC, HPMEC and HUVEC) (**Figures 2A, C, E, G**, respectively). The IRBC-endothelial binding was significantly lower when OSF-2 was supplied (Paired t-test: *t* = 12.60, *df* = 5, *P* < 0.0001 for hCMEC/D3; *t* = 20.17, *df* = 5, *P* < 0.0001 for HRGEC; *t* = 7.652, *df* = 5, *P* = 0.0006 for HPMEC*; t* = 8.92, *df* = 5, *P* = 0.0003 for HUVEC) (**Figures 2B, D, F, H**, respectively).

**Figure 3:**
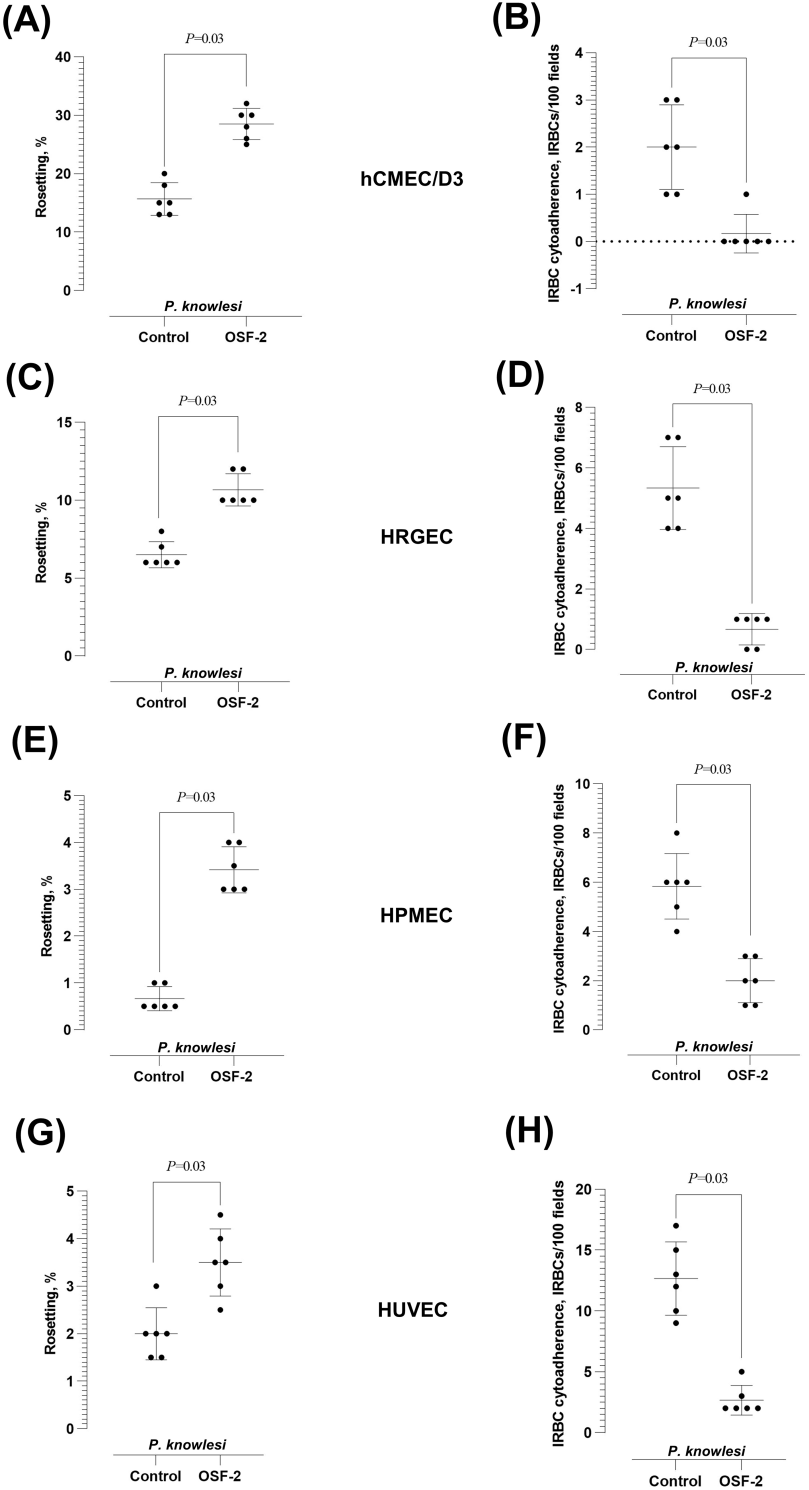
Effect of OSF-2 on the *P. knowlesi-*IRBC cytoadherence dynamics between URBC and endothelial cells. **(A, B)** Rosetting rates **(A)** and IRBC-endothelial cytoadherence rates **(B)** from the experiments with hCMEC/D3. **(C, D)** Rosetting rates **(C)** and IRBC-endothelial cytoadherence rates **(D)** from the experiments with HRGEC. Horizontal dotted line in **(C)** represents zero value (the detection limit of the assay). **(E, F)** Rosetting rates **(E)** and IRBC-endothelial cytoadherence rates **(F)** from the experiments with HPMEC. **(G, H)** Rosetting rates **(G)** and IRBC-endothelial cytoadherence rates **(H)** from the experiments with HUVEC. Error bars in the plots represent mean and S.D.

Similar OSF-2-mediated effect was observed with *P. knowlesi* when tested with hCMEC/D3 (**Figures 3A, B**), HRGEC (**Figures 3C, D**), HPMEC (**Figures 3E, F**), and HUVEC (**Figures 3G, H**). The rosetting rates by the zoonotic parasite were significantly increased by OSF-2 (Wilcoxon matched-pairs signed rank test *W* = 21; *P* = 0.03 for hCMEC/D3, HRGEC, HPMEC and HUVEC) (**Figures 3A, C, E, G**, respectively), whereas the *P. knowlesi*-IRBC-endothelial cytoadherence was significantly reduced under OSF-2-supplied settings (Wilcoxon matched-pairs signed rank test *W* = 21; *P* = 0.03 for hCMEC/D3, HRGEC, HPMEC and HUVEC) (**Figures 3B, D, F, H**, respectively). Of note, at basal level, the *P. falciparum* IRBC-endothelial binding rate was the highest with the brain-derived hCMEC/D3, and the *P. knowlesi* IRBC-endothelial binding rate was the highest with the umbilical cord-derived HUVEC. To sum up, following the coincubation of OSF-2, IRBC, URBC, and endothelial cells, the protein significantly increased the rosetting rates while reducing the IRBC-endothelial cytoadherence with endothelial cell lines derived from the brain, kidney, lung, and umbilical cord.

## 4 Discussion

In this study, we characterized OSF-2 as a novel host-derived factor that facilitates type II rosette formation by *P. falciparum* and *P. knowlesi*. To date, other host-derived factors, including IGFBP7, TSP-1, VWF, naturally occurring anti-band 3 Ig G, CFD and Ig M, have been reported to promote type II rosette formation (Clough et al., 1998; Rowe et al., 2002; Luginbühl et al., 2007; Lee et al., 2020, 2022). OSF-2 exerts its rosette-stimulatory effect at concentrations (150 ng/ml for *P. knowlesi*, and 200 ng/ml for *P. falciparum*) that are consistent with those reported in pathological conditions (**Supplementary Table 2**) (Zhu et al., 2016; Ding et al., 2018). Of note, the range of OSF-2 concentrations tested in this study were based on various non-malaria pathological conditions. Therefore, it is imperative to investigate the serum OSF-2 level of malaria patients with different disease severity in future.

The OSF-2-mediated rosette-stimulation is serum-dependent, suggesting that other serum components are needed to facilitate the rosette-stimulation induced by OSF-2. Notably, the presence of human serum is also crucial for the IRBC surface expression of cytoadherence ligands by *P. falciparum* (Langreth et al., 1979). The importance of serum in the cytoadherence of *P. knowlesi*-IRBC has also been documented (Lee et al., 2022c). However, it is important to emphasize that, in this study, we observed that both species of parasites could form rosettes under serum-free conditions (**Figures 1C, D**), albeit of lower rosetting rates. This highlights the complexity of rosetting in *P. falciparum* and *P. knowlesi*, where different types of rosettes have varying formation requirements. In addition, it is important to note that the basal rosetting rates by the IRBC were relatively low. We did not perform any procedure to concentrate the rosette-forming IRBC prior to the experiment, so that the non-rosetting IRBC in basal condition were covered in our investigations.

Various host-derived proteins have been deciphered as the receptors that interact with the IRBC (Lee et al., 2022b), including the blood group A and B antigens that have been investigated intensively in the field of malaria cytoadherence research (Carlson and Wahlgren, 1992; Chotivanich et al., 1998; Barragan et al., 2000; Vigan-Womas et al., 2012; Hedberg et al., 2021). Here, we ruled out the involvement of human A or B blood group antigens in the OSF-2-mediated rosetting for *P. falciparum* and *P. knowlesi*. Nevertheless, more in-depth investigations are needed to decipher the actual receptors involved in the OSF-2-mediated rosetting. Besides, the effect of OSF-2 on the expression profile of various cytoadherence receptors by the endothelial cells deserves to be investigated further. With regards to the parasite-derived ligands involved in OSF-2-mediated rosetting, PfEMP1 is likely the involving *P. falciparum*-derived ligands. Among the discovered *P. falciparum* rosetting ligands, PfEMP1 is highly sensitive to trypsin treatment (removed from the surface of the IRBC after treatment of 10 µg/mL of trypsin), whereas ligands like RIFIN and STEVOR are partially resistant to trypsin treatment (require higher concentration of trypsin to be cleaved from the surface of the IRBC) (Fernandez et al., 1999; Kyes et al., 2000; Chan et al., 2014; Niang et al., 2014; Bachmann et al., 2015; Goel et al., 2015; Lee et al., 2020). Our approach with trypsin treatment on the purified IRBC ruled out the involvement of STEVOR and RIFIN as the ligands of OSF-2-mediated rosetting. Importantly, these experiments did not place PfEMP1 as the only parasite-derived IRBC surface protein that is involved in the OSF-2-mediated cytoadherence. The cytoadherence ligands of *P. knowlesi* have yet to be deciphered. Nevertheless, the *P. knowlesi*-derived ligands involved in OSF-2-mediated rosetting are highly sensitive to trypsin treatment.

From the basal IRBC-endothelial binding assays, both species of malaria parasites demonstrated varied binding affinity to different human endothelial cells. As demonstrated earlier (Lee et al., 2022c), *P. knowlesi* had low affinity to the brain-derived hCMEC/D3 (**Supplementary Figure 4A**) as compared to other endothelial cell types, whereas *P. falciparum* demonstrated the highest binding affinity to hCMEC/D3 among the human endothelial cell lines tested (**Supplementary Figure 4B**). Interestingly, *P. knowlesi*-IRBC showed the highest binding affinity to the umbilical cord-derived HUVEC. Although *P. knowlesi* infections during pregnancy are rare (Barber et al., 2015), an experimental infection of *P. knowlesi* on pregnant baboons revealed the accumulation of parasites in the placenta (Onditi et al., 2015). Besides, the binding interaction between *P. knowlesi*-IRBC and HUVEC has been documented previously (Chuang et al., 2022).

As mentioned earlier, PfEMP1 mediates both the rosetting and IRBC-endothelial binding by *P. falciparum*. In fact, IRBC with dual cytoadherence properties have been reported (Kaul et al., 1991; Udomsangpetch et al., 1992; Adams et al., 2014). Nevertheless, the dynamics and equilibrium of these cytoadherence events remained to be elucidated fully. Here, we demonstrated that the presence of OSF-2 skewed the IRBC cytoadherence tropism towards the URBC (via rosette formation), as compared to the endothelial cells. Given that IRBC-endothelial cytoadherence can lead to endothelial activation and injury, the reduced tendency of IRBC to interact with endothelial cells under the presence of OSF-2 may alter the pathogenesis route of malaria, hence the disease outcome of the infection. OSF-2 may interact with the cytoadherence players (the parasite-derived ligand and the host-derived receptors), and alter the cytoadherence outcomes by either hampering the interaction between the ligand and endothelial-specific receptors, or facilitating rosette formation between IRBC and URBC, hence hampering the binding of IRBC to endothelial cells due to steric hindrance. Nevertheless, more studies are needed in future to decipher the mechanisms of OSF-mediated changes to the *Plasmodium*-IRBC cytoadherence dynamics. Besides, the OSF-2-mediated IRBC cytoadherence changes should be investigated further to evaluate its potential application as an adjunct treatment regime to reduce the manifestation of severe malaria. Taken together, we reported the involvement of OSF-2 as a novel host-derived factors of type II rosetting and characterized the role of OSF-2 in the IRBC cytoadherence dynamics between URBC and endothelial cells for *P. falciparum* and *P. knowlesi*.

## Data Availability

The original contributions presented in the study are included in the article/**supplementary material**. Further inquiries can be directed to the corresponding author.
