# Supplementary material for: The role of periostin (OSF-2) in the cytoadherence phenomena mediated by malaria parasites
Source: Front Cell Infect Microbiol. 2025 May 13;15:1599872. doi: 10.3389/fcimb.2025.1599872 (PMC12106375; doi:10.3389/fcimb.2025.1599872)
Supplement: Supplementary file 1 [file DataSheet1.pdf]

Supplementary Material

Supplementary table 1. Information on tools, reagents and biological materials used in this study.

Reagent/Resource	Reference or source	Identifier/ Catalog Number / Notes
**Biological materials**		
A1-H.1 (*Plasmodium knowlesi*)	Laboratory-adapted parasite strain; maintained in Universiti Malaya and A*STAR ID labs.	Used for all experiments.
3D7 (*Plasmodium falciparum*)	Laboratory-adapted parasite strain; maintained in Universiti Malaya and A*STAR ID labs.	Used for all experiments. Parasites derived from three distinct culture cycles contributed three biological replicates in experiments for Figures 1A, 1C, 1E, 1G. Parasites derived from two distinct culture cycles contributed two biological replicates in experiments for Figures 2A-H.
NF54 (*Plasmodium falciparum*)	Laboratory-adapted parasite strain; maintained in Universiti Malaya	Used for all experiments. Parasites derived from two distinct culture cycles contributed two biological replicates in experiments for Figures 1A, 1C, 1E, 1G and Figures 2A-H.
FVT402 (*Plasmodium falciparum*)	Laboratory-adapted parasite strain; maintained in Universiti Malaya and A*STAR ID labs.	Used for all experiments. Parasites derived from three distinct culture cycles contributed three biological replicates in experiments for Figures 1A, 1C, 1E, 1G. Parasites derived from two distinct culture cycles contributed two biological replicates in experiments for Figures 2A-H.
hCMEC/D3 (human cerebral microvascular endothelial cells)	A kind gift of Pierre Olivier Couraud, Institut Cochin, Paris, France; maintained in Universiti Malaya and A*STAR ID labs	Used for all cytoadherence assay.
HPMEC (human pulmonary microvascular endothelial cells)	ScienCell™ Research Laboratories; maintained in Universiti Malaya and A*STAR ID labs	Cat# 3000; used for all cytoadherence assay.
HRGEC (human renal glomerular endothelial cells)	ScienCell™ Research Laboratories; maintained in Universiti Malaya and A*STAR ID labs	Cat# 4000; used for all cytoadherence assay.
HUVEC (human umbilical vein endothelial cells)	A kind gift of A*STAR ID labs; maintained in Universiti Malaya	Used for all cytoadherence assay.
**Chemicals, Enzymes and other reagents**		
1X phosphate buffer saline (PBS)	Gibco™	Cat#20012-027
AlbuMAX II™	Gibco™ ThermoFisher Scientific	Cat#11021-037
D-glucose	Sigma-Aldrich®	Cat#G7520-1KG
Dimethyl sulfoxide (DMSO)	Sigma-Aldrich®	Cat#D2650
Endothelial cell medium (ECM) set	ScienCell™ Research Laboratories	Cat#1001
Fetal Bovine Serum (FBS)	ScienCell™ Research Laboratories	Cat#0025
Gelatin solution	Sigma-Aldrich®	Cat#G1393-100ml
Giemsa	Sigma-Aldrich®	Cat#48900-500ML-F
Human B serum	-	-
Hypoxanthine	Sigma-Aldrich®	Cat#H9377-25G
Immersion oil	Systerm	Cat#IM372-90
L-Glutamine	Sigma-Aldrich®	Cat#G8540-100G
Methanol	Friendemann Schmidt	Cat#M2097-4-4001
Periostin/OSF-2 (50µg)	R&D Systems	Cat#3548-F2
Phosphate buffered saline tablets	Sigma	Cat#S26-36
RPMI 1640 medium	Gibco™	Cat#23400-013-1L
Sodium bicarbonate	Sigma-Aldrich®	Cat#S5761-500G

Sodium chloride	J.T.Baker	Cat#3624-69
Sodium hydroxide	SYNERLAB	Cat#SY-CS10237
StemPro™ Accutase™ cell dissociation reagent	ThermoFisher Scientific	Cat#A1110501
Trypan blue	Sigma-Aldrich®	Cat#T6146
Trypsin solution	Sigma-Aldrich®	Cat# T3924-100ml
**Software**		
GraphPad Prism version 9.5.1	GraphPad	
**Others**		
0.5-10µl micropipette tips	Kirgen	Cat#KG5131-L
1.5ml microcentrifuge tube	GSBIO	Cat#CC102-N-F
1000µl micropipette filtered tips	Axygen	Cat#TF-1000-R-S
15ml Falcon tube	Biofil	Cat#CFT021500
200µl micropipette filtered tips	Axygen	Cat#TF-200-L-R-S
25cm^2 cell culture flask canted neck (plug-seal cap)	SORFA	Cat#210110
3ml sterile transfer pipette	Biologix	Cat#30-0138A1
500ml vacuum bottle filter	Biofil	Cat#Fpe204500
50ml Falcon tube	Biofil	Cat#CFT01115
50mL sterile syringe	Terumo	Cat#SS*50LE
75cm^2 cell culture flask canted neck (plug-seal cap)	SORFA	Cat#210200
96-well microplates	Nunclon Surface	Cat#137101
Cellulose acetate syringe filter (0.22um pore size)	Bioflow Lifescience	Cat#MALCA25022
Cryovial	Corning	Cat#430488
Falcon® Cell Culture Flask T25, filter cap	VWR™	Cat#29185298
Glass slide	Sail brand	Cat#7101
Kimwipes	Kim Tech Science Brand	Cat#34155/34120
Lab-tek chamber slide w/ cover permanox slide sterile- 8 wells	Thermo Scientific Nunc	Cat#1383823
LABTEK chamber slides	ThermoFisher Scientific	Cat#177445
LD columns	Miltenyi Biotec	Cat#130-042-901
Microscope coverslip 22x22mm	Mariendfeld	Cat#0101052
Microscope slide with frosted side	Citoglas	Cat#P/N.0312-2101
Parafilm	Bemis	Cat#PM-996
QuadroMACS™ separator	Miltenyi Biotec	Cat#130-090-976
Vacutainer	BD	Cat#367284

Supplementary table 2. Range of serum OSF-2 levels in various physiologic and pathological conditions.

**References**	**Physiologic serum level (ng/mL)**	**Pathological serum level (ng/mL)**
Gadermaier *et al.*, 2018	Median: 78.5<br>Range: 36.1 to 133.3	N/A
Caswell-Smith *et al.*, 2016	Median: 50.1<br>Range: 28.1 to 136.4	N/A
Yildiz *et al.*, 2021	61.4 ± 5.13	Exacerbated asthma<br>101.6 ± 32.8
Yang *et al.*, 2016	75.96	Nonalcoholic fatty liver disease<br>126.75
Ninomiya *et al*., 2018	Median: 87.5<br>Range: 28 to 245	Eosinophilic Chronic Rhinosinusitis<br>Mild:<br>Median: 104.5<br>Range: 54 to 259<br><br>Moderate:<br>Median: 114<br>Range: 40 to 325<br><br>Severe:<br>Median: 136<br>Range: 48 to 369
Kou *et al*., 2024	N/A	Atopic dermatitis<br>144
Thuwaji *et al.*, 2017	N/A	Cholangiocarcinoma<br>>94
Yamaguchi *et al.*, 2013	N/A	Systemic sclerosis<br>104.9
Okamoto *et al.*, 2011	N/A	Idiopathic interstitial pneumonias<br>107.1±11.9
Zhu *et al.*, 2016	N/A	Non-small cell lung cancer<br>1914.16
Ding *et al.*, 2017	N/A	Diabetic retinopathy<br>1670.20
Tuna *et al*., 2024	27.4 ± 14.1	COVID-19 with macrophage activation syndrome (MAS): 43.9 ± 20<br>COVID-19 without MAS 37.2 ± 22

Supplementary figures

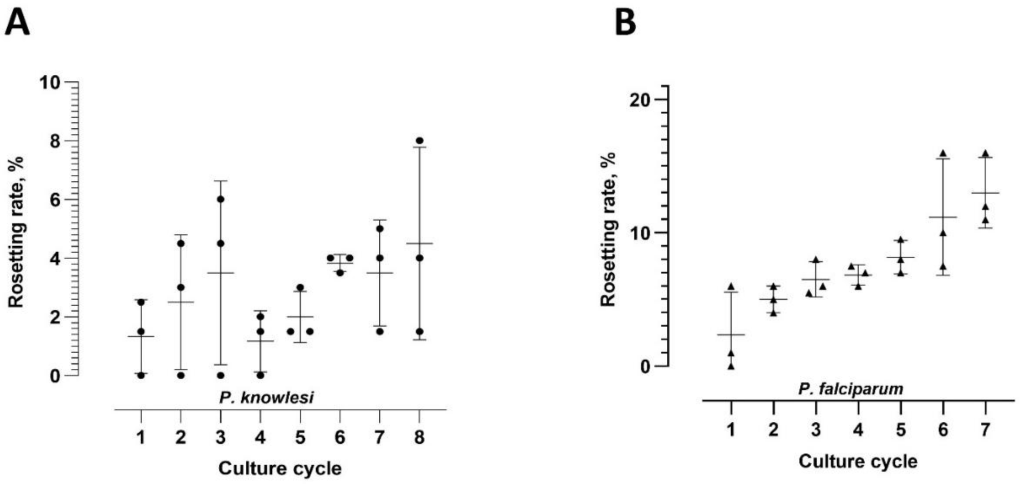

Supplementary figure 1. Basal rosetting rates of *P. knowlesi* (A) and *P. falciparum* (B) across continuous cycles of cultivation. Three biological replicates were performed for each experiment.

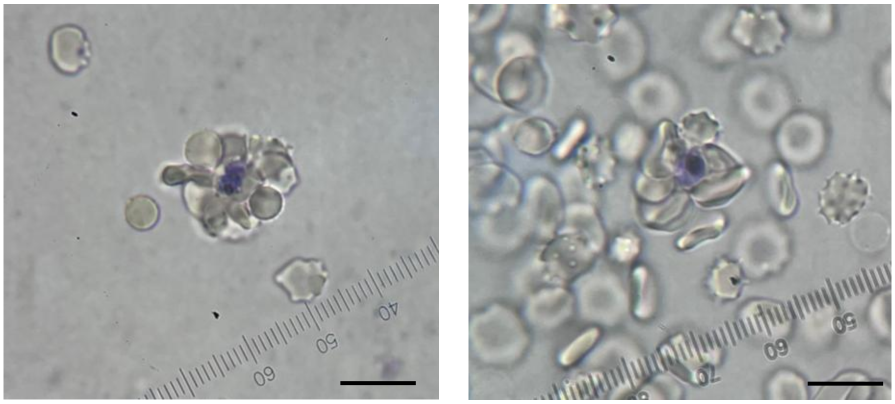

Supplementary figure 2. Rosettes formed by *P. knowlesi* (left) and *P. falciparum* (right). Wet mount preparation from 5% Giemsa staining, 1,000 X magnification. Scale bars represent 10 mm.

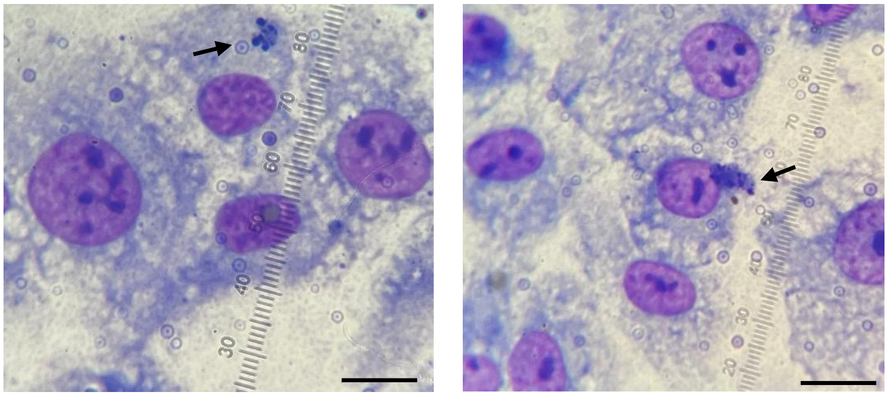

Supplementary figure 3. *Plasmodium* IRBC–endothelial cytoadherence. Snap shots of *P. knowlesi* IRBC (arrows) adhered to HUVEC primed with culture supernatant of *P. knowlesi* culture 1,000 X magnification. Scale bars represent 10 mm.

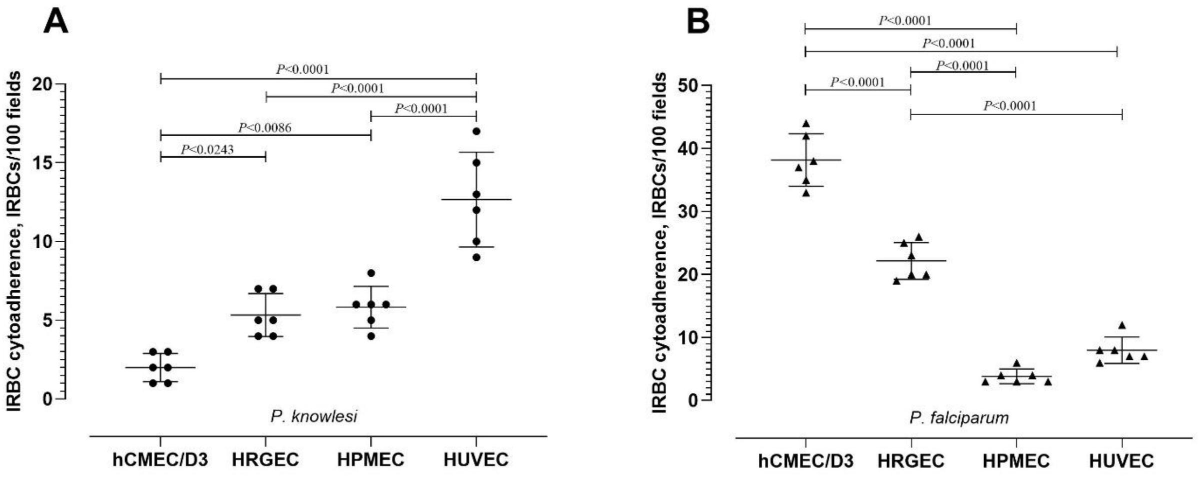

Supplementary figure 4. Comparison of IRBC–endothelial cytoadherence by *P. knowlesi* (A) and *P. falciparum* (B) with different human endothelial cell lines. One-way ANOVA with Tukey's test was performed. For *P. knowlesi*, the basal IRBC-HUVEC binding was significantly higher than those of HPMEC, HRGEC and hCMEC/D3. For *P. falciparum*, the basal IRBC-hCMEC/D3 binding was significantly higher than those of HRGEC, HPMEC, and HUVEC.
